# The Year-Book of Charity

**Published:** 1921-01-29

**Authors:** 


### The Hospital

The Workers' Newspaper of Administrative Medicine and Institutional Life. Administration. National Insurance and Health.

No. 1808, Vol. LXIX. SATURDAY, JANUARY 29, 1921. PRICE TWOPENCE

### THE YEAR-BOOK OF CHARITY.

In view of the death of its founder, it is satisfactory to see "Burdett's Hospitals and Charities" still making its annual reappearance, and to learn that arrangements are being made to regain, in regard to the date of its appearance, some of the time lost during the war. Some improvements have, we think, already been made in the new edition. The sections devoted to hospitals, Poor-law infirmaries, dispensaries, and asylums now follow each other. The convalescent homes have been grouped under the general charities' section, and are succeeded by tuberculosis sanatoria. The list of periodicals and the form of bequests have been taken to the end of the volume. We are also glad to see the index in the forefront, so that it is the first thing to meet the eye when the pages of the preface have been turned. We note, too, that the preliminary chapters have been condensed; and, if this means that the tables must be studied more closely, it avoids a repetition in words of facts which are more easily understood in figures. Moreover, Chapter XII., "Hospital Finance and Expenditure in 1918," has been split into two. All the tables relating to "beds, patients and comparative costs" are placed together, and the "analysis of expenditure under all heads" is likewise set by itself. A more rigid revision, we think, could improve the directory, but the information is so extensive, hard to check, and from new institutions often so difficult to obtain, that we must be grateful for all that we have before we complain of any omission. If hospital authorities would make the book better known by taking pains to acquaint themselves better with its varied contents, they would render the public and the Editor invaluable aid. At any rate each could secure that the information concerning his own institution is complete and up-to-date, and that it arrives within a few days of the receipt of the form requesting the particulars. The posting of a second or third request for information adds greatly to the heavy cost which the production of such a work must entail. Hospitals should be proud of their own work of reference, or at least see that it shall err through no fault of their own.

The volume remains unique upon its subject, and since that is mainly statistical, the pages chiefly invite the attention of those who are in search of information not accessible in a digested form anywhere else. We can recommend to the general reader, however, that section of the Preface in which the Editor seeks to explain a much debated problem. In 1918, the last year of the war, the voluntary hospitals possessed a surplus of total income over total expenditure "amounting to £746,008." Why, then, was their position so serious in 1920? There have been various attempts to explain this. No two people seem to have been agreed. It is therefore interesting to quote the explanation offered in "Burdett's Hospitals and Charities." "In the first place," we read, "the excess of income, though large in the aggregate, was not equally distributed. Secondly, large sums for deferred expenditure on structural alterations and cleaning are now demanded. Beside this, the capitation grants for the treatment of soldiers have been withdrawn with their departure, so that a considerable source of income, peculiar to the war period, has disappeared. Next, the improvement in the hours and salaries of nurses has inflated the wages bill, and meant an increased staff and additional accommodation, all of which send up the cost of maintenance and necessitate heavy new expenses in the domestic service. These changes in the aggregate, with rising prices, help to explain the rapid alteration in the financial position," and how it is that the present difficulties arose.

This statement, at least, is succinct and definite. We commend it to the general newspapers and particularly to the *Pall Mall Gazette*, whose columns a year ago gave so alarming an account of the voluntary hospitals' "bankruptcy." In justice to the founder of this volume, let us remember that a year ago, when the voluntary system was supposed to be at the end of its resources, the late Sir Henry Burdett remained unshaken in his belief that it would survive the peril. His buoyancy and reiteration positively annoyed those who were suffering from the scare; some of these had at least the excuse that their responsibilities were enormous. With such hospital chairmen and treasurers we, and Sir Henry at the time, sincerely sympathised. But it was necessary for *some one* to remind those weighed down with responsibilities, and still more the general public, that the voluntary system was not dying, and that its elasticity was great. Sir Henry Burdett did this, and a year later we can test the truth of his words.

In the past year of despair and difficulty the great Funds have made such progress as would have seemed astonishing even in prosperous times. King Edward's Hospital Fund for London raised upwards of £190,000. The Hospital Sunday Fund total of £121,058 has created a record, whilst the Hospital Saturday Fund's receipts show an increase of over £30,000 on the previous year, which itself was a record amount. The League of Mercy has raised upwards of £22,000, excluding £4,000 from the British Red Cross, against £21,519 last year.

Of course, the difficulties are not over. They never will be over, for life is a battle which is never won. As the victories succeed each other a new standard is created. The criterion of success is simply the capacity to go on living. Our point here is that the year 1920, which was regarded by the pessimists as their own peculiar property, has proved less disheartening than it might have been. The workings of the voluntary system which has achieved this result are shown in "Burdett's Hospitals and Charities," and we believe that its author would have found in the new volume an excellent justification for the faith which he expressed a year ago.

